# The lipidome of primary murine white, brite, and brown adipocytes—Impact of beta-adrenergic stimulation

**DOI:** 10.1371/journal.pbio.3000412

**Published:** 2019-08-01

**Authors:** Sabine Schweizer, Gerhard Liebisch, Josef Oeckl, Marcus Hoering, Claudine Seeliger, Carolin Schiebel, Martin Klingenspor, Josef Ecker

**Affiliations:** 1 ZIEL—Institute for Food & Health, Technical University of Munich, Freising, Germany; 2 Chair of Molecular Nutritional Medicine, TUM School of Life Sciences, Technical University of Munich, Freising, Germany; 3 EKFZ—Else Kröner-Fresenius Center for Nutritional Medicine, Technical University of Munich, Freising, Germany; 4 Institute of Clinical Chemistry and Laboratory Medicine, University Hospital Regensburg, Regensburg, Germany; Rockefeller University, UNITED STATES

## Abstract

Lipid species patterns are conserved within cells to maintain physicochemical properties of membranes and cellular functions. We present the lipidome, including sterols, glycerolipids (GLs), glycerophospholipids (GPLs), and sphingolipids (SLs), of primary ex vivo differentiated (I) white, (II) brite, and (III) brown adipocytes derived from primary preadipocytes isolated from (I) epididymal white, (II) inguinal white, and (III) intrascapular brown adipose tissue. Quantitative lipidomics revealed significantly decreased fractions of phosphatidylcholine (PC) and phosphatidylethanolamine (PE), with longer (C > 36) and more polyunsaturated species, as well as lower levels of cardiolipin (CL) in white than in brite and brown adipocytes. Together, the brite and brown lipidome was comparable and indicates differences in membrane lipid packing density compared with white adipocytes. Changes in ceramide species profile could be related to the degree of browning. Beta-adrenergic stimulation of brown adipocytes led to generation of saturated lyso-PC (LPC) increasing uncoupling protein (UCP) 1-mediated leak respiration. Application of stable isotope labeling showed that LPC formation was balanced by an increased de novo synthesis of PC.

## Abstract 2

Quantitative lipidomics reveal that the cell membrane lipidome of brite and brown adipocytes is comparable, but significantly different to that of white adipocytes. Beta-adrenergic stimulation of brown adipocytes induces generation of saturated lysophosphatidylcholine from phosphatidylcholine, increasing uncoupling protein (UCP) 1-mediated leak respiration.

## Introduction

Mammalian adipose tissue (AT) can be categorized in white and brown AT. Adipocytes from white AT (WAT) store excess energy in the form of triacylglycerides (TGs) that can be released as free fatty acids (FAs) into the circulation when necessary. They represent globular cells with a single large lipid droplet [1]. Brown adipocytes contain several small lipid droplets and can generate heat to maintain a stable body temperature by nonshivering thermogenesis, which is mediated by uncoupling protein 1 (UCP1) [2]. In addition to white and brown adipocytes, mice and humans harbor inducible brown, also known as brite or beige, adipocytes [3–5]. Specifically in inguinal WAT (iWAT), brite adipocytes can appear in response to cold exposure, providing an extra thermogenic capacity [6,7].

The lipid composition of cell membranes significantly influences generic physical membrane parameters, including lipid packing density and fluidity [8]. Cholesterol has a planar structure and intercalates between phospholipid chains. It promotes lipid packing and the transition of membranes from a fluid to solid gel phase. As cholesterol, phospholipids containing saturated acyl chains pack with higher density forming less-fluid bilayers, whereas monounsaturated fatty acyls having a kinked shape reduce packing density and increase fluidity [9]. Polyunsaturated acyl chains can switch easily between different conformations facilitating rapid membrane conversions, such as endocytosis or vesicle formation [10].

We and others have previously shown that the cell membrane lipid composition is precisely controlled, cell type-specific, and adapted to cellular functions [11,12]. Lipidomic data of total white and brown AT are available [13–16]. Cellular lipidomes are only available for cell lines 3T3L1 (white adipocytes) and BAT-C1 (brown adipocytes) [17].

Thus, the aim of the present study was to investigate the lipidomes of primary white, brite, and brown adipocytes. Terminally differentiated adipocytes were generated from preadipocytes isolated from epididymal white AT (eWAT), iWAT and intrascapular brown AT (iBAT) of 129SV/S6 mice. Quantitative mass spectrometry–based lipidomics showed that white adipocytes (eWAT) significantly differed from brite (iWAT) and brown adipocytes (iBAT) in their lipid class and species profile. Further, we identified that the β-adrenergic agonist isoproterenol (ISO) induces generation of lysophosphatidylcholine (LPC) from phosphatidylcholine (PC) in brown adipocytes leading to alteration of mitochondrial bioenergetics.

## Results

### Adipocytes derived from murine eWAT, iWAT, and iBAT show marker pattern characteristic for white, brite, or brown adipocytes

**Fig 1:**
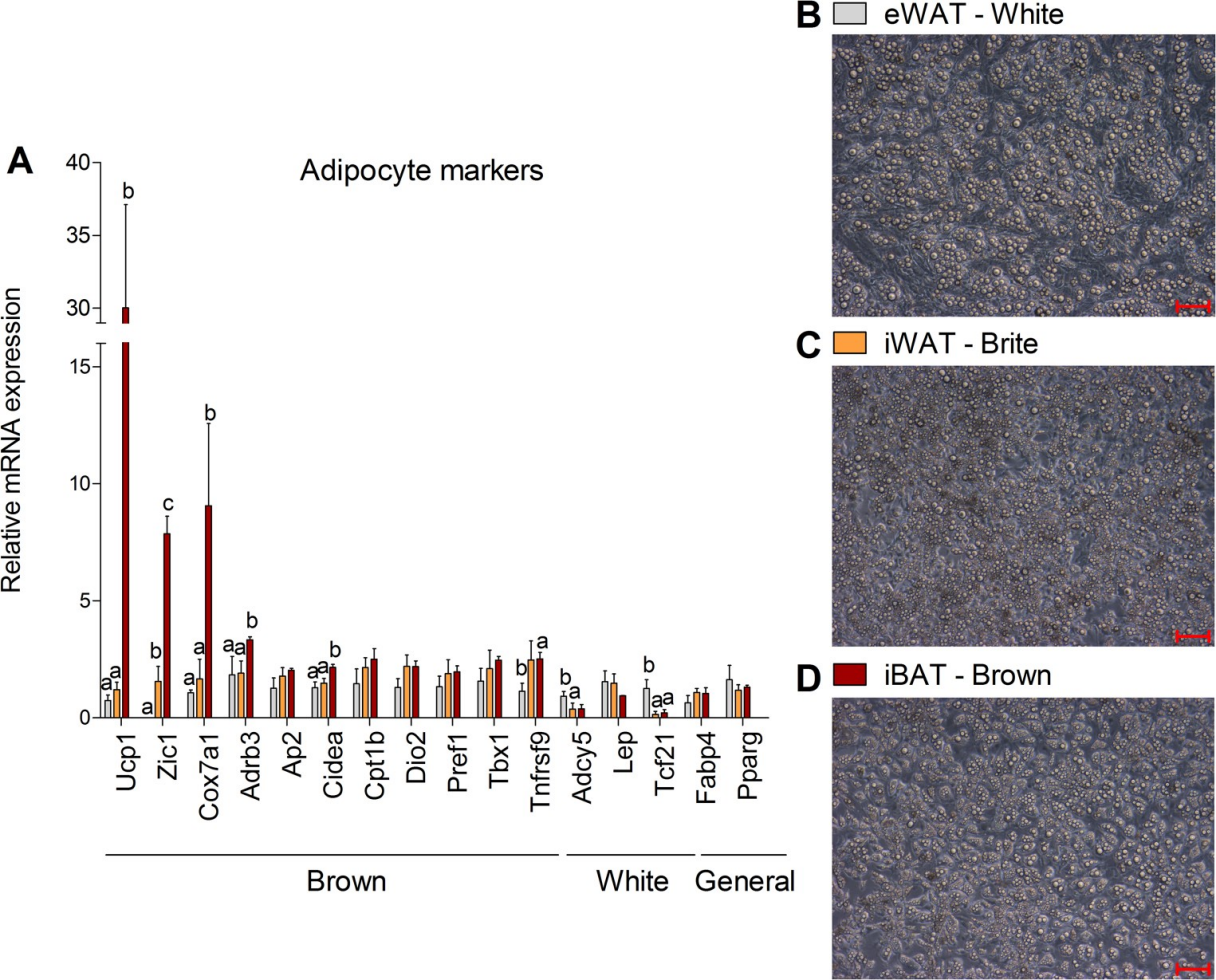
Characterization of eWAT, iWAT, and BAT derived adipocytes. (A) Expression profile of genes that have been linked previously to white or brown adipocytes, WAT or BAT, or AT browning. Shown are means ± SD of 3 mice; annotation of “a, b” indicates that group "a" is statistically different from "b"; annotation of “a, b, c” indicates that all 3 groups are significantly different from each other; significant difference was tested using a one-way ANOVA (Post Hoc: Tukey Test; *p* < 0.05). (B–D) Representative images show eWAT, iWAT, and BAT derived adipocytes; the “bright dots” are lipid droplets; images are scaled equally; the red line equals 100 μm. The underlying data of (A) can be found in S1 Data. BAT, brown adipose tissue; iBAT, intrascapular brown adipose tissue; eWAT, epididymal white adipose tissue; iWAT, inguinal white adipose tissue; WAT, white adipose tissue.

Preadipocytes, isolated from eWAT, iWAT, and iBAT of 129SV/S6 mice, were differentiated ex vivo to adipocytes. Cells from eWAT and iBAT differentiate into homogenous populations of white and brown adipocytes. Preadipocytes derived from iWAT, which are in this study referred as “brite” adipocytes, are a heterogeneous population of brite and white fat cells. This is confirmed by mRNA expression analysis of genes that were previously related to specific adipocyte types, AT depots, or AT browning [7,18–20]. Transcription of Ucp1, Zic1, Cox7a1, and most other brown adipocyte markers was highest brown adipocytes (iBAT) followed by brite (iWAT) and white adipocytes (eWAT; Fig 1A). Expression levels of genes characteristic for white adipocytes were comparable in iWAT-derived cells to either those of eWAT (Lep) or iBAT (Adcy5, Tcf21), arguing for their mixed phenotype. Comparable transcript levels of general adipocyte markers (Fig 1A) and amounts of lipid droplets (Fig 1B–1D) indicate that the different adipocyte populations were equally differentiated.

### Glycerophospholipid, sphingolipid, and glycerolipid composition of white differs from brite and brown adipocytes

To test whether the different cell types differ in the lipidome composition, we quantified cellular glycerophospholipids (GPLs), sphingolipids (SLs), glycerolipids (GLs), and sterols using direct infusion electrospray ionization coupled to tandem mass spectrometry (ESI-MS/MS) and high-resolution mass spectrometry (HR-MS). Our analyses comprised the following:

- Lipids present in all cell membranes (common membrane lipids) including phosphatidyl-choline (PC; GPL), lysophosphatidyl-choline (LPC; GPL), phosphatidylethanolamine (PE; GPL), PE-based plasmalogens (PE P; GPL), phosphatidylinositol (PI; GPL), phosphatidylserine (PS; GPL), sphingomyelin (SM; SL), ceramide (Cer; SL), hexosylceramide (HexCer; SL), and free cholesterol (FC; sterol).
- Cardiolipin (CL; GPL), predominantly located in inner mitochondrial membranes [8].
- Storage lipids including cholesterylester (CE; sterol), diacylglycerol (DG; GL), and triacylglycerol (TG; GL).

**Fig 2:**
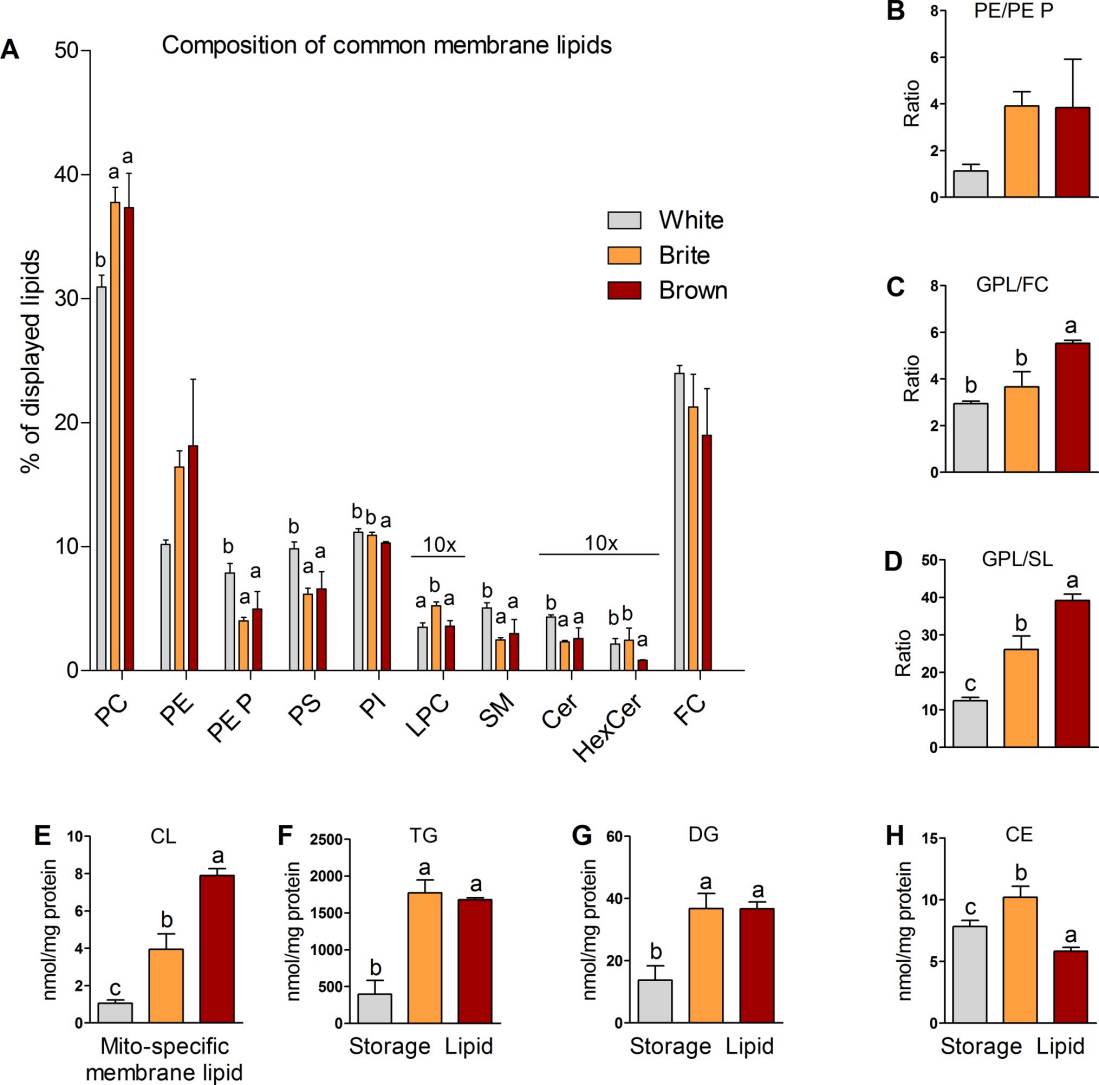
Lipid class composition of white, brite, and brown adipocytes. (A) Common membrane lipids, (B) PE/PE P, (C) GPL/FC, (D) GPL/SL, (E) CL, (F) TG, (G) DG, (H) CE. Shown are means ± SD of 3 independent experiments, each performed in triplicates with AT pooled from 3 mice; annotation of “a, b” indicates that group "a" is statistically different from "b"; annotation of “a, b, c” indicates that all 3 groups are significantly different from each other; significant difference was tested using a one-way ANOVA (Post Hoc: Tukey Test; *p* < 0.05). The underlying data of (A–H) can be found in S1 Data. CE, cholesterylester; Cer, ceramide; CL, cardiolipin; DG, diacylglycerol; FC, free cholesterol; GPL, glycerophospholipid; HexCer, hexosylceramide; LPC, lyso-PC; PC, phosphatidylcholine; PE, phosphatidylethanolamine; PE P, PE-based plasmalogen; PI, phosphatidylinositol; PS, phosphatidylserine; SL, sphingolipid; SM, sphingomyelin; TG, triacylglycerol.

With approximately 35%, PC was the dominating lipid class of common membrane lipids followed by FC with approximately 21%, PE with approximately 15%, and PI with approximately 11% (Fig 2A, S1A Fig). The lipid class composition of brite and brown adipocytes was comparable and differs significantly from that of white adipocytes. White adipocytes contained less PC and PE but higher proportions of PS and PE P than brite and brown cells. Whereas approximately 50% of ethanolamine-containing lipids were PE P in white, brite and brown adipocytes contained only approximately 20% PE P fraction (Fig 2B). Levels of CL were greatest in brown adipocytes (Fig 2E). The GPL/FC ratio was lower in white (ratio = 3) compared with brite and brown adipocytes (ratio = 3.7 and 5.5; Fig 2C). The GPL/SL ratio was approximately 2 to 3 times higher in brite and brown adipocytes (Fig 2D) because the fractions of Cer and SM were significantly decreased in these cells (Fig 2A). The dominating storage lipid was TG, followed by DG and CE (Fig 2F–2H; both with at least approximately 30-fold lower levels); TG and DG concentrations were approximately 3 times higher in brite and brown than white adipocytes.

### Brite and brown adipocytes have largely comparable PC, PE, and PI species profiles

**Fig 3:**
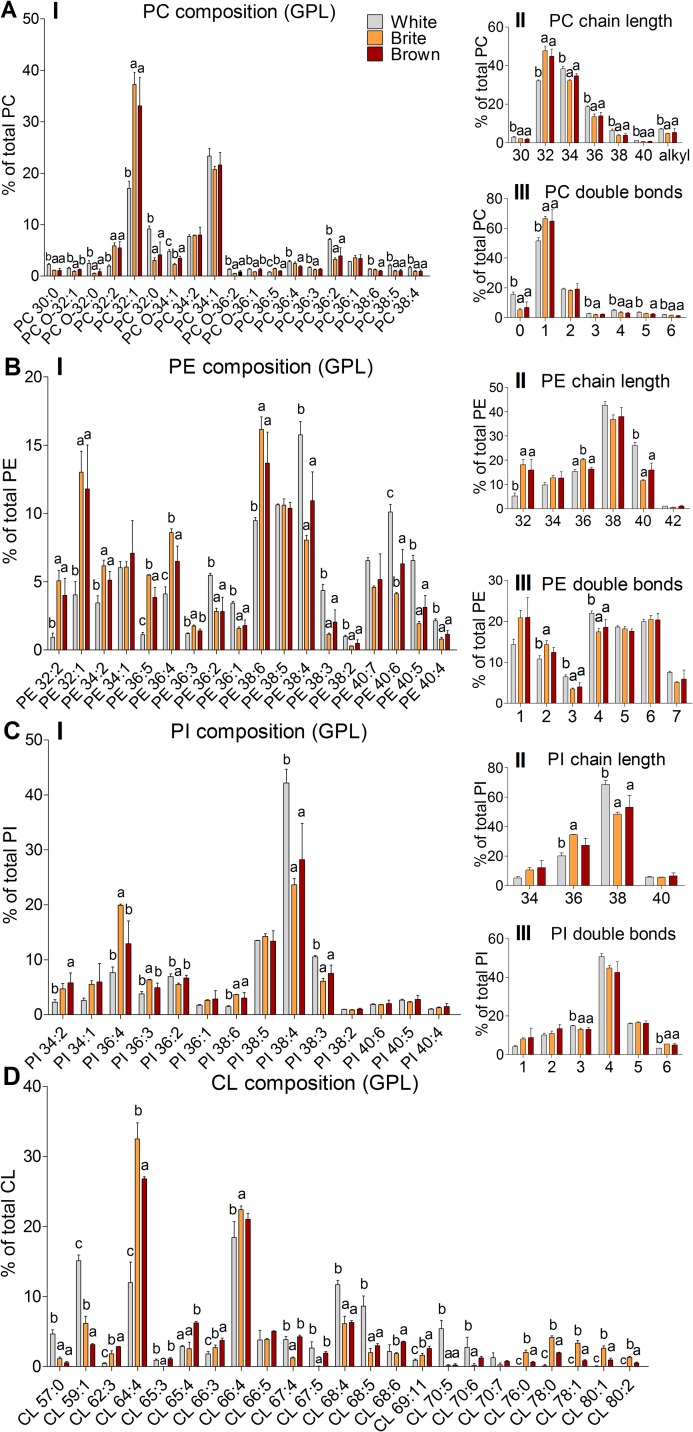
Lipid species profiles of major GPL from white, brite, and brown adipocytes. (A) PC, (B) PE, (C) PI, (D) CL. Panels are (I) species composition, (II) chain length as the number of carbons in the sum of FA moieties, and (III) saturation as the number of double bonds in the sum of FA moieties. Shown are means ± SD of 3 independent experiments, each performed in triplicates with AT pooled from 3 mice; annotation of “a, b” indicates that group "a" is statistically different from "b"; annotation of “a, b, c” indicates that all 3 groups are significantly different from each other; significant difference was tested using a one-way ANOVA (Post Hoc: Tukey Test; *p* < 0.05). The underlying data of (A–D) can be found in S1 Data. AT, adipose tissue; CL, cardiolipin; FA, fatty acid; GPL, glycerophospholipid; PC, phosphatidylcholine; PE, phosphatidylethanolamine; PI, phosphatidylinositol.

Next, we asked whether the species profile of the major GPL classes is specific for the adipocyte cell type. For all cells, the major PC species were monounsaturated PC 32 and 34 and di-unsaturated PC 32 through 36 species (Fig 3A). PE acyl chains were generally longer and more unsaturated than PC (Fig 3B). Major PI species were PI 36:4 and PI 38:4 (Fig 3C). The LPC species profile was dominated by saturated and monounsaturated C16 and C18 species (S1B Fig).

Comparing the individual cell types, we found a systematic shift from longer and polyunsaturated GPL species in white adipocytes to shorter and monounsaturated GPL species in brite and brown adipocytes. Proportions of PC, PE, and PI species containing carbons >36 (Fig 3A–3C, II) and double bonds >2 (Fig 3A–3C, III) were higher in white adipocytes—including PC 36:2, PC 38:4, and PC 38:5; PE 36:2, PE 38:4, PE 40:4, and PE 40:5; and PI 38:3 and PI 38:4 (Fig 3A–3C, I). In contrast, the species patters of brite and brown adipocytes were rather enriched with shorter (carbons: 32–34) and monounsaturated acyl chain containing GPL, including PC 32:1 and PE 32:1. The alkyl-containing PC (PC O) fraction was approximately 2 times higher in white than in brite and brown adipocytes (Fig 3A, II). As for PC, the LPC species profile was comparable between brite and brown adipocytes but different to white adipocytes (S1B Fig). White adipocytes contained higher amounts of PUFA-containing species such as LPC 20:4 and LPC 22:6. However, as CL levels (Fig 2E), the CL species pattern was also significantly different between all 3 adipocyte types (Fig 3D).

### Brite adipocytes differ in their PS profile (GPL) from white and brown adipocytes

**Fig 4:**
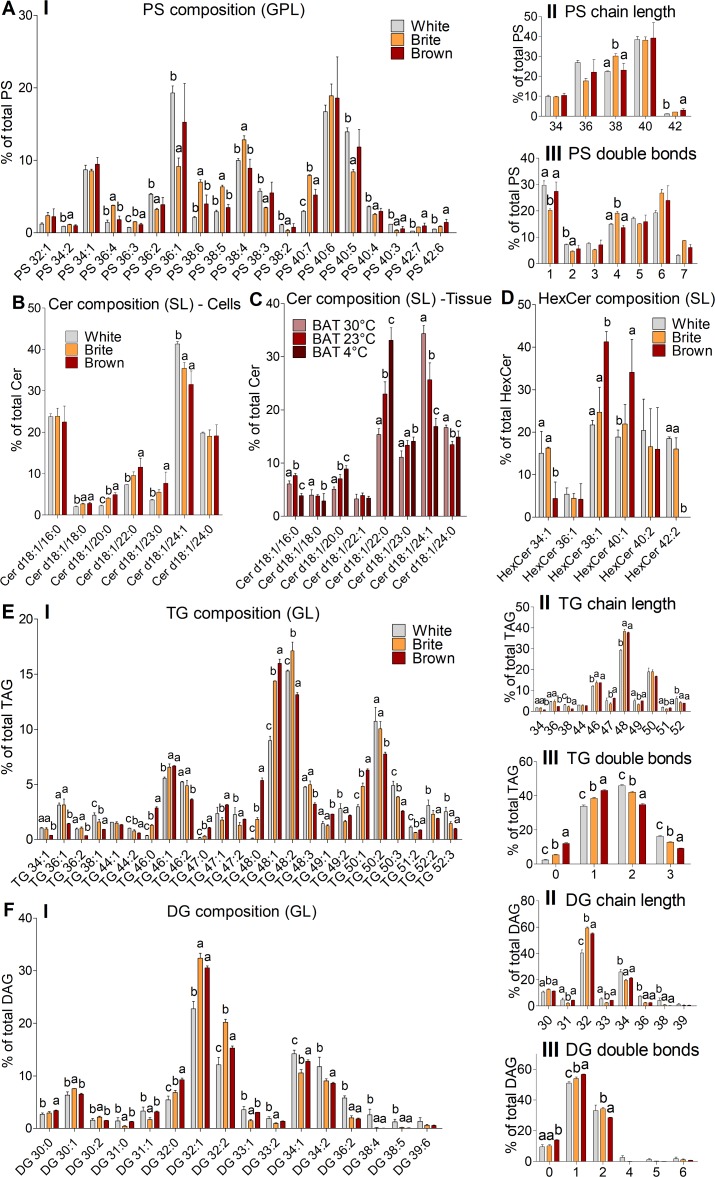
PS (GPL), Cer and HexCer (SL), TG and DG (GL) species composition of white, brite, and brown adipocytes; Cer (SL) profile of BAT at different browning degrees. (A) PS, (B) Cer in adipocytes; (C) Cer in BAT samples from mice housed for 7 days at 4°C, 23°C, or 30°C; (D) HexCer, (E) TG, (F) DG in adipocytes. Panels show (I) species composition, (II) chain length as the number of carbons in the sum of FA moieties, (III) saturation as the number of double bonds in the sum of FA moieties. For adipocytes (A–B, D–F) means ± SD of 3 independent experiments are shown, each performed in triplicates with AT pooled from 3 mice; for BAT (C), the means ± SD of *n* = 9 (4°C), *n* = 9 (23°C), and *n* = 12 (30°C) mice are shown; annotation of “a, b” indicates that group "a" is statistically different from "b"; annotation of “a, b, c” indicates that all 3 groups are significantly different from each other; significant difference was tested using a one-way ANOVA (Post Hoc: Tukey Test; *p* < 0.05). The underlying data of (A–F) can be found in S1 Data. Cer, ceramide; BAT, brown adipose tissue; DG, diacylglycerol; FA, fatty acid; GL, glycerolipid; GPL, glycerophospholipid; HexCer, hexosylceramide; PS, phosphatidylserine; SL, sphingolipid; TG, triacylglycerol.

Surprisingly, white and brown adipocytes were rather similar in their PS species profiles and differed from that of brite adipocytes. Brite cells were significantly enriched in PS species with 38 carbons and polyunsaturated species containing 4 double bonds (Fig 4A, II-III) including PS 38:4 (Fig 4A, I). Less unsaturated PS species with 1 or 2 double bonds, such as PS 36:1 and PS 38:2, were enriched in white and brown cells (Fig 4A, I).

### Cer species (SL) relate to adipocyte andAT browning status

Analysis of the Cer profiles revealed increasing fractions of Cer d18:1 with saturated acyl chains from C20 to C23 but decreasing contents of d18:1/24:1 in brown adipocytes compared with white adipocytes, suggesting a systematic change according to the adipocyte browning status (Fig 4B). To provide further evidence for this hypothesis, 129SV/S6 mice were housed at 4°C, 23°C, and 30°C for 7 days to promote or antagonize AT browning. Cer profiling in BAT samples showed that d18:1/24:1 significantly dropped with the housing temperature (30°C > 23°C > 4°C), whereas saturated C20 to C23 species increased (Fig 4C). These results clearly indicate that the Cer profile, particularly the species 20:0, 22:0, and 24:1, correlates with AT browning.

We did not observe related changes in the SM species composition (S1C Fig). HexCer 38:1 and 40:1 were higher in brown than in white and brite fat cells (Fig 4D). TG and DG pattern were for the most part different between all 3 cell types (Fig 4E).

### β-Adrenergic stimulation of brite and brown adipocytes induces generation of LPC

Next, we asked whether lipolysis induction by β-adrenergic stimulation affects the membrane lipidome. Fractions of the major GPL classes of adipocytes treated with ISO (0.5 μM, 0.5 hours) did not change significantly, except for an increase of the LPC fraction in brite and brown adipocytes (S2A and S2B Fig).

**Fig 5:**
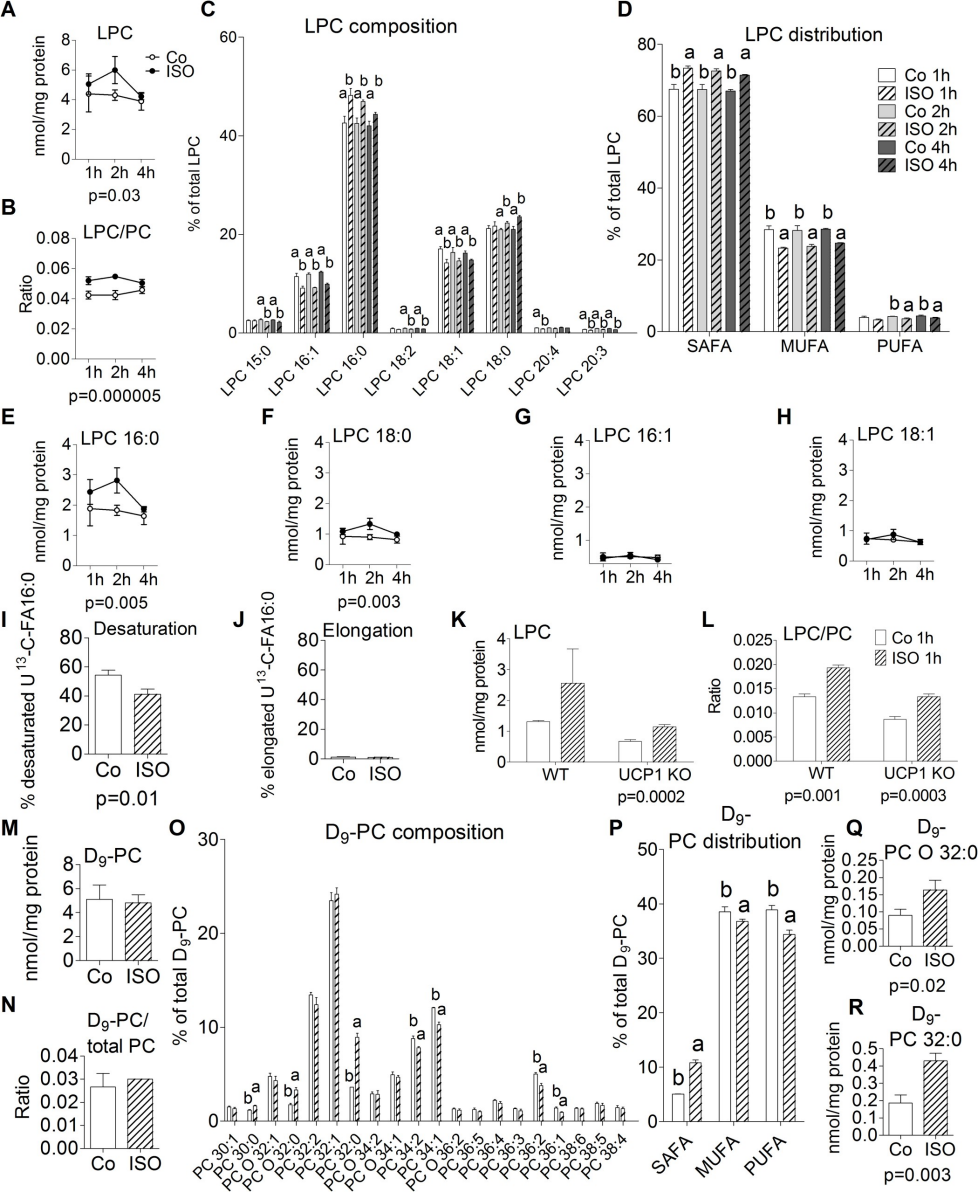
Effects of β-adrenergic stimulation in brite adipocytes. (A) LPC, (B) LPC/PC, (C) LPC composition, (D) LPC distribution, (E) LPC 16:0, (F) LPC 18:0, (G) LPC 16:1, (H) LPC 18:1; primary cells were treated with 0.5 μM ISO for 1 hour, 2 hours, or 4 hours. (I) U-^13^C-palmitate to–palmitoleate desaturation, (J) U-^13^C-palmitate to–stearate elongation; primary cells were simultaneously treated with 0.5 μM ISO and 100 μM U-^13^C-pamitate for 1 hour. (K) LPC, (L) LPC/PC; primary cells originating from UCP1 KO mice and WT littermates stimulated for 1 hour with 0.5 μM ISO. (M) D_9_-PC (N) D_9_-PC/total PC, (O) D_9_-PC composition, (P) D_9_-PC distribution, (Q) D_9_-PC 32:0, (R) D_9_-PC O-32:0; cells were simultaneously incubated with D_9_-choline and 0.5 μM ISO for 1 hour. Shown are means ± SD of 3 mice, the *p*-value indicates a significant difference between the treatment groups “Co” and “ISO”; annotation of “a, b” indicates that group "a" is statistically different from "b," which was determined for panels (A–B) and (E–H) using a two-way ANOVA (Post Hoc: Tukey Test) and was determined for (C–D) and (I–R) using a Student *t* test. The underlying data of (A–R) can be found in S1 Data. Co, control; ISO, isoproterenol; KO, knock out; LPC, lyso-PC; MUFA, monosaturated fatty acid; PC, phosphatidylcholine; PUFA, polyunsaturated fatty acid; SAFA, saturated fatty acid; UCP, uncoupling protein; WT, wild type.

To verify these results and to investigate the dynamics of this LPC increase, brite adipocytes were treated with ISO for 1 hour, 2 hours, and 4 hours. We found that ISO treatment elevated total LPC levels by approximately 15% to 39% (0.7–1.7 nmol/mg) and the LPC/PC ratio approximately 24% to 28% at 1 to 2 hours (Fig 5A and 5B). Analysis of the LPC species profile and distribution revealed that ISO-induced the generation of saturated LPC species, including LPC 16:0 and 18:0 (Fig 5C and 5D). At 1 to 2 hours of β-adrenergic stimulation, LPC 16:0 and LPC 18:0 levels were increased by 30% to 54% (0.6–1.0 nmol) and 17% to 48% (0.1–0.4nmol), whereas monounsaturated LPC 16:1 and 18:1 levels were unaffected (Fig 5E–5H). This might be due to an altered desaturation capacity because 1 hour of ISO treatment significantly lowers desaturation of U-^13^C- palmitate/FA16:0 to U-^13^C-palmitoleate/FA16:1 *n-7* by approximately 25% (Fig 5I). Cellular elongation capacity (U-^13^C- stearate/FA 18:0 generation) was very low compared with desaturation (Fig 5J).

To test whether the generation of LPC upon β-adrenergic stimulation depends on UCP1 activity, experiments with cells from UCP1 knockout (KO) mice were performed [21]. ISO treatment led to elevated LPC levels and LPC/PC ratios in brite and brown UCP1 KO adipocytes and corresponding wildtype (WT) cells (Fig 5K and 5L, S2C–S2L Fig). These results demonstrate that LPC generation after β-adrenergic stimulation is independent of UCP1.

Next, we tested whether ISO-mediated generation of LPC impacts PC de novo synthesis via the Kennedy Pathway by D_9_-choline incorporation. Therefore, we analyzed D_9_-PC generation after ISO stimulation for 1 hour in brite adipocytes. We could neither observe differences in total D_9_-PC levels nor in the D_9_-PC/total PC ratio between control and ISO treated cells (Fig 5M and 5N). But analysis of the D_9_-PC composition and distribution showed that β-adrenergic stimulation significantly induced the synthesis of saturated PC 32 species (Fig 5O and 5P), i.e., D_9_-PC 32:0 and D_9_-PC O-32:0 (Fig 5Q and 5R).

### LPC 16:0 enhances UCP1-mediated leak respiration in brown adipocytes after β-adrenergic stimulation

Finally, we asked whether modulation of cellular LPC content affects mitochondrial bioenergetics. Therefore, brown adipocytes were incubated with LPC 16:0 (5 and 25 μM) for 1 hour prior to acquisition of the following mitochondrial bioenergetics profiles using microplate-based respirometry: (1) basal respiration; (2) inhibition of ATP synthase by oligomycin (Oligo) to distinguish oxygen consumption (coupled respiration) used for ATP synthesis from the basal proton leak (basal uncoupled respiration); (3) activation of UCP1 by addition of ISO to determine the UCP1-mediated leak respiration; (4) assessment of maximal respiratory capacity by using carbonyl cyanide 4-(trifluoromethoxy) phenylhydrazone (FCCP) as uncoupling agent; (5) blocking the electron transport chain with antimycin A (Anti A) to leave only the nonmitochondrial oxygen consumption.

**Fig 6:**
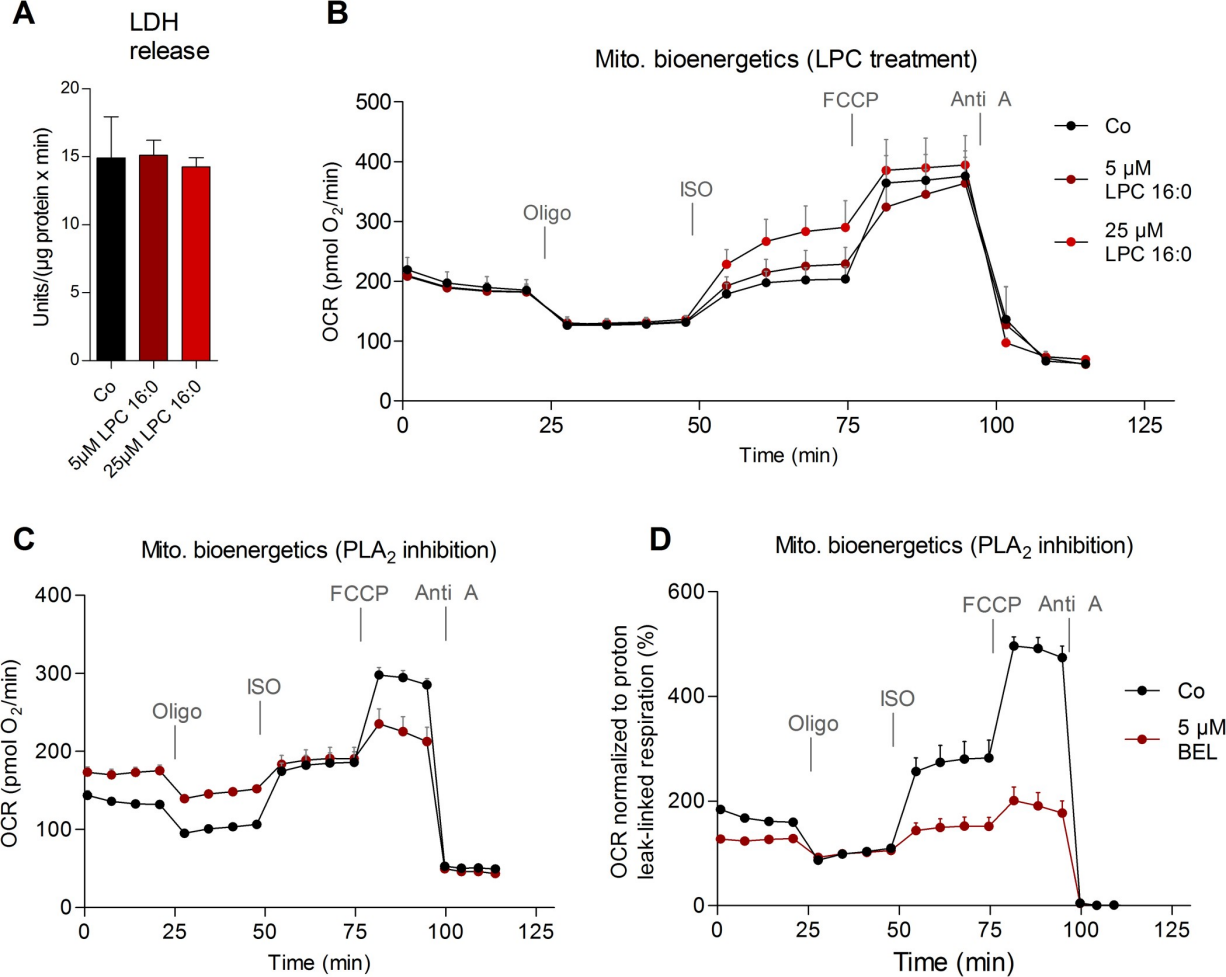
Mitochondrial bioenergetics in brown adipocytes after treatment with LPC 16:0 or BEL. (A) LDH release after stimulation with 5 μM and 25 μM LPC 16:0 for 1 hour. Shown are means ± SD of triplicates with AT pooled from 3 mice. (B) Mitochondrial bioenergetics profile (OCR in pmol O_2_/min), including basal respiration, basal leak respiration, UCP1-mediated leak respiration, maximal oxygen consumption, and nonmitochondrial respiration of primary brown adipocytes after stimulation with 5 μM and 25 μM LPC 16:0 for 1 h. Shown are means ± SEM of *n* = 7 (Co, 5 μM LPC) and *n* = 8 (25 μM LPC) with AT pooled from 3 mice. Mitochondrial bioenergetics—(C) OCR shown in pmol O_2_/min and (D) OCR baselined to basal leak respiration—of brown adipocytes pretreated with 5 μM BEL for 1 hour. Shown are means ± SEM of *n* = 14 (Co) and *n* = 13 (BEL) with AT pooled from *n* = 3 mice. The underlying data of (A–D) can be found in S1 Data. Anti A, antimycin A; AT, adipose tissue; BEL, bromoenollactone; Co, control; FCCP, carbonyl cyanide 4-(trifluoromethoxy) phenylhydrazone; ISO, isoproterenol; LDH, lactate dehydrogenase; LPC, lyso-PC; Mito,; OCR, oxygen consumption rate; Oligo, oligomycin; PLA_2_, phospholipase A_2_; UCP, uncoupling protein.

The LPC 16:0 doses applied were not cytotoxic as determined by monitoring the release of lactate dehydrogenase (LDH; Fig 6A). We found that ISO-induced UCP1-mediated leak respiration was increased in LPC 16:0 treated brown adipocytes (Fig 6B). Further, bromoenollactone (BEL), an inhibitor of calcium-independent phospholipase A_2_ blocking LPC generation and PC remodeling [22], enhanced basal respiration (Fig 6C) but inhibited ISO-induced UCP1-dependent oxygen consumption (Fig 6D). These results indicate that modulation of cellular LPC levels affects brown adipocyte function, including UCP1-mediated respiration after β-adrenergic stimulation.

## Discussion

The aim of the present study was to provide lipidomic data of primary white (eWAT), brite (iWAT), and brown (BAT) adipocytes generated on a single platform to allow a systematic comparative evaluation of their lipidomes. The different cell types were characterized by the mRNA expression profile of established marker genes or transcripts that were previously related to white or brown adipocytes, white or brown AT, or AT browning [7,18,20,23]. Cells had access to essential polyunsaturated FAs (PUFAs), because the cultivation media contains fetal bovine serum (FBS) comprising comparable FA species and amounts as typically found in human plasma (S1 Table).

We found that white adipocytes can be differentiated from brite and brown cells based on both their GPL composition and species profiles of the major phospholipid classes. White adipocytes had lower amounts of PC and PE, and their acyl chains were longer and more unsaturated. Brite and brown adipocytes were rather enriched in shorter and monounsaturated species. Using semiquantitative mass spectrometry–based profiling of 3T3L1 preadipocytes, differentiated 3T3L1 adipocytes, and BAT-C1 cells (brown adipocytes), a previous study reported differences for a few selected lipid species [17]. A systematic difference according to chain length and the degree of saturation as identified in our study was not described. Using tandem mass spectrometry (MS/MS) approaches based on quadrupole or time of flight mass spectrometry, previous studies presented lipidomic data for total mouse tissue samples including mouse gonadal WAT (comparable to eWAT in our study), subcutaneous WAT (comparable to iWAT in our study), and BAT (summarized in S2 Table) [13,14,16]. Part of our results is in agreement with these findings, including higher CL levels in BAT and brown adipocytes than in subcutaneous WAT (sWAT) and brite adipocytes [13]. However, although we found no difference between total brite and brown adipocyte DG and TG concentrations, higher levels were reported in sWAT than in BAT [14,16].

The GPL/FC ratio was highest in brown and lowest in white adipocytes. This might be explained by the cell morphology and organelle content because brown adipocytes contain a high amount of mitochondria with low amounts of cholesterol [8,24]. Brite adipocytes are somewhere in between brown and white with mixed morphologic and organelle characteristics [5], which is clearly reflected in their content of CL located in mitochondria. Further, we observed that white adipocytes had a significantly higher amount of PE P (approximately 2-fold) than brite and brown adipocytes. PE P are known as cellular antioxidants because of their ability to bind free radicals [25]. Production of reactive oxygen species (ROS) is increased in WAT of obese mice and cultured adipocytes exposed to oxidative stress [26]. Lower levels of PE P in brown adipocytes may be related to UCP1 activity, which decreases ROS generation [27]. Concerning membrane organization, the sn1 vinyl-ether linkage of PE P permits tighter packing of the membrane [28,29]. Overall, together with a decreased GPL/FC ratio and a significantly increased fraction of saturated PC species, it could be hypothesized that white adipocytes, which primarily store lipids, have more rigid and tighter packed membranes compared with brown adipocytes, which are the metabolically more active cells.

Analysis of the Cer species profiles in white, brite, and brown adipocytes and BAT samples from mice housed at different temperatures demonstrate that AT browning correlates with Cer d18:1 species 20:0, 22:0, and 24:1. This supports the recent finding that adipocyte total Cer levels regulate subcutaneous adipose browning in C57BL/6J mice and that Cer synthesis is a critical factor for mitochondrial function of brite adipocytes [30]. Moreover, it fits with the concept that bioactive ceramide exerts species-specific effects [31].

Upon β-adrenergic stimulation in ATs lipolysis of TG to free FA by adipose triglyceride lipase (ATGL) and hormone-sensitive lipase (HSL) is induced [32]. Free FA are required for UCP-1 activation and related heat production [33]. We identified that PC breakdown is also affected. These results are supported by the finding that rats injected with 40 mg/kg ISO for 24 hours have significantly increased myocardial levels of LPC but decreased levels of PC [34]. LPC generation was inhibited if rats were pretreated with phospholipase inhibitors (e.g., chlorpromazine) or β-adrenergic receptor blockers (propranolol). Experiments with brite and brown UCP1 KO adipocytes revealed that PC degradation is independent of UCP1 activity. Further, we suggest that saturated LPC 16:0 and 18:0 species are generated from saturated PC 32 species after ISO stimulation due to a reduced desaturase activity.

Surprisingly, recent data generated with KO mice having a loss of brown adipose-specific ATGL or comparative gene identification-58 (CGI-58; coactivator of ATGL) function demonstrated that TG degradation in BAT is not a prerequisite for cold-induced thermogenesis because FA released from WAT can activate UCP1 [35–37]. Based on our lipidomic data set and the finding that treatment of cells with BEL—a Group VI PLA_2_ inhibitor—prevents ISO-induced UCP1-mediated respiration, we propose that an alternative model for UCP1 activation might be free FA release from PC. This is in line by the observation that FA liberated from the inner mitochondrial membrane by PLA_2_ can activate UCP1 [38]. It has to be clarified in further studies which PLA_2_ isoform might be relevant to explain these and our findings. BEL inhibits the Group VI PLA_2_ superfamily, which comprises at least 6 members with multiple subtypes in humans and mice with largely unknown phospholipid class and FA specificity [39,40].

We propose that in addition to the free FA, LPC affects UCP1 activity because LPC 16:0 enhances UCP1-mediated leak respiration after β-adrenergic stimulation in brown adipocytes. Serum LPC 16:0 levels of healthy men were positively correlated with BAT activity monitored with cold-induced [^18^F] fluorodeoxyglucose ([^18^F] FDG) positron emission tomography–computed tomography (PET-CT) strongly supporting our findings [41].

In summary, white, brite, and brown primary adipocytes are characterized by distinct lipidomes, which reflect their different cell functions and organelle composition—features that are also mirrored in the extent of LPC generation upon β-adrenergic stimulation. Investigation of the complex and dynamic lipidomic architecture on the cellular level is perquisite to understand pivotal adipocyte functions. These include (I) energy storage, which is closely associated patho-physiological conditions such as obesity or metabolic syndrome; (II) signaling processes occurring at cell membrane surfaces, which depend on physicochemical membrane properties and thus the membrane lipid class and acyl-chain composition; and (III) fundamental cellular functions such as thermogenesis and mitochondrial respiration in brown adipocytes. In this study, the detailed and precise characterization of the cellular lipidome identified saturated LPC as potential regulator of UCP1 activity and Cer species as markers for AT browning.

## Materials and methods

### Ethics statement

The breeding and experimental use of mice used for this study was reviewed and approved by the local institution in charge (Regierung von Oberbayern; approval numbers: 55.2-1-54-2531-99-13-2015, 55.2-1-54-2532-17-2015, and 55.2-1-54-2532-34-2016).

### Mouse housing and cell culture experiments

For primary cell cultures, 5- to 6-week-old male 129SV/S6 mice fed a chow diet (R/M-H, Ssniff, Soest, Germany) were used. Adipocyte precursor cells were isolated from fat tissues as previously described by Li and colleagues [23]. For studies employing UCP1 KO cells, adipocyte precursor cells were isolated from 5- to 6-week-old UCP1 KO (129S1/SvImJ) mice and corresponding WT littermates [21]. Experiments were performed with primary (Fig 4K and 4L, S2C and S2D Fig) and immortalized cells (S2E–S2L Fig) generated with retrovirus-delivered SV40 large T antigen (pBABE-puro SV40 LT, supplied by Ronald Kahn) [42].

After reaching confluency induction medium (10% FBS (Biochrom, Berlin, Germany); 0.5 mM isobutylmethylxanthin, 125 nM indomethacin, 1 mM dexamethasone, 850 nM insulin, 1 nM T3 (Sigma Aldrich, Taufkirchen, Germany);, 1 μM rosiglitazone (Biomol, Hamburg, Germany)) was added for 2 days. Subsequently, cells were maintained in differentiation medium (850 nM insulin, 1 nM T3, 1 μM rosiglitazone) for 8 days. Medium was changed every 2 days.

For PC metabolic labeling studies, cells were incubated with 50 μg/ml D_9_-choline chloride in choline-free medium obtained from Cambridge Isotope Laboratories (Tewksbury, Massachusetts). To investigate palmitate metabolism, cells were incubated with 100 μM U^13^-C-palmitate coupled with FA-free bovine serum albumin (BSA; molar ratio free FA/BSA: approximately 1/1).

For BAT browning studies, 10-week-old male 129SV/S6 mice fed a chow diet (R/M-H, Ssniff, Soest, Germany) were housed at 4°C, 23°C, and 30°C for 7 days.

### RNA isolation and quantitative real-time PCR analysis

Total RNA was extracted from cells using the RNEasy Mini Kit (Qiagen, Hilden, Germany). The purity and integrity of the RNA were assessed using the Agilent 2100 bioanalyzer (Agilent Technologies, Waldbronnn, Germany). For real-time PCR (RT-PCR), 2 μg RNA was transcribed into cDNA using the Reverse Transcription System from Promega (Mannheim, Germany). Quantitative RT-PCR analysis was performed using the Light Cycler LC 480 (Roche, Mannheim, Germany). Transcription factor II B (Tf2b) was used as a reference gene. Relative quantification was carried out using the LightCycler 480 SW 1.5.1 (Roche, Mannheim, Germany). The following primers were used: UCP1 (fw: ʹ3-GTACACCAAGGAAGGACCGA-5ʹ; rev: ʹ3-TTTATTCGTGGTCTCCCAGC-5ʹ), zinc finger protein of the cerebellum 1 (Zic1; fw: ʹ3-AACCTCAAGATCCACAAAAGGA-5ʹ; rev: ʹ3-CCTCGAACTCGCACTTGAA-5ʹ), cytochrome c oxidase subunit 7A1 (Cox7a1; fw: ʹ3-GCCGACAATGACCTCCCAGTA-5ʹ; rev: ʹ3- TGTTTGTCCAAGTCCTCCAA-5ʹ), adrenergic receptor beta 3 (Adrb3; fw: ʹ3-CAGCCAGCCCTGTTGAAG-5ʹ; rev: ʹ3-CCTTCATAGCCATCAAACCTG-5ʹ), transcription factor AP-2 alpha (Tfap2a; fw: ʹ3-TACTGCGGAGAGCGAAGTCTAA-5ʹ; rev: ʹ3-AGCTTTACGTCTCCCTGCTGG-5ʹ), cell death-inducing DNA fragmentation factor alpha subunit-like effector A (Cidea; fw: ʹ3-TGCTCT TCTGTATCGCCCAGT-5ʹ; rev: ʹ3-GCCGTGTTAAGGAATCTGCTG-5ʹ), carnitine palmitoyltransferase 1b (Cpt1b; fw: ʹ3-GGCACCTCTGGGAGTTTGTCCT-5ʹ; rev: ʹ3-TGCTCGGGAATGTCCCAC-5ʹ), deiodinase iodothyronine type II (Dio2; fw: ʹ3-GATGCTCCCAATTCCAGTGT-5ʹ; rev: ʹ3-TGAACCAAAGTTGACCACCA-5ʹ), Pref1/delta-like noncanonical Notch ligand 1 (Dkl1; fw: ʹ3-CTGCGAAATAGACGTTCGGGCTT-5ʹ; rev: ʹ3-TGTGCTGGCAGTCCTTTCC-5ʹ), T-box 1 (Tbx1; fw: ʹ3-GGCAGGCAGACGAATGTTC-5ʹ; rev: ʹ3-TTGTCATCTACGGGCACAAAG-5ʹ), tumor necrosis factor receptor superfamily member 9 (Tnfrsf9; fw: ʹ3-CGTGCAGAACTCCTGTGATAAC-5ʹ; rev: ʹ3-GTCCACCTATGCTGGAGAAGG-5ʹ), adenylate cyclase 5 (Adcy5; fw: ʹ3-CATCTCTCTGCACACCAACT-5ʹ; rev: ʹ3-GCAGGAGAAGATGAGGACA-5ʹ), leptin (Lep; fw: ʹ3-CAGGATCAATGACATTTCACACA-5ʹ; rev: ʹ3-GCTGGTGAGGACCTGTTGAT-5ʹ), transcription factor 21 (Tcf21; fw: ʹ3-CATTCACCCAGTCAACCTG-5ʹ; rev: ʹ3-TTCCTTCAGGTCATTCTCTGG-5ʹ), FA binding protein 4 (Fabp4; fw: ʹ3-GATGGTGACAAGCTGGTGGT-5ʹ; rev: ʹ3-TTTATTTAATCAACATAACCATATCCA-5ʹ), Peroxisome proliferator activated receptor gamma (Pparg; fw: ʹ3-GAAGGATGCAAGGGTTTTTTCC-5ʹ; rev: ʹ3-GGCACTTCTGAAACCGACAGT-5ʹ), transcription factor II B (Tf2b; fw: ʹ3- TGGAGATTTGTCCACCATGA-5ʹ; rev: ʹ3-GAATTGCCAAACTCATCAAAACT-5ʹ).

### Mitochondrial bioenergetics

Mitochondrial bioenergetics were assayed using microplate-based respirometry [21]. Oxygen consumption rate (OCR) was measured at 37°C using an XF96 Extracellular Flux Analyzer (Agilent Technologies, Waldbronn, Germany). Brown preadipocytes were seeded in XF96 cell culture microplates and differentiated as described before. On day 6 of differentiation, cells were washed twice with preheated assay medium (unbuffered DMEM supplemented with 25 mM glucose (Sigma Aldrich, Taufkirchen, Germany) and 2 mM Glutamax (Fisher Scientific, Loughborough, UK)) and incubated in assay medium supplemented with 2% essentially FA-free BSA (Sigma Aldrich, Taufkirchen, Germany) for 1 hour at 37°C in a non-CO_2_ incubator prior to the measurement. Basal respiration was measured in untreated cells, ATP-linked respiration was inhibited by Oligo (5 μM), UCP1-dependent oxygen consumption was recorded after the addition of ISO (0.5 μM), maximum oxidative capacity was measured after FCCP (7 μM) stimulation, and nonmitochondrial oxygen consumption was detected following the injection of Anti A (5 μM) (Sigma Aldrich, Taufkirchen, Germany). For BEL treatments (Biomol, Hamburg, Germany), mitochondrial bioenergetics were monitored without BSA in the culture media to prevent association with BEL impacting its cellular delivery and efficiency [43]. Therefore the doses of ISO and FCCP had to be adjusted appropriately to 2.5 nM and 1 μM. LPC 16:0 was dissolved in culture media, and cytotoxicity was tested by monitoring LDH activity in the culture medium by measuring the consumption of NADH at the absorbance of 339 nm necessary for the conversion of pyruvate to lactate.

### Lipidomics

Lipids were extracted according to the procedure described by Bligh and Dyer in the presence of non-naturally occurring lipid species as internal standards [44]. The following lipid species were added as internal standards: PC 14:0/14:0, PC 22:0/22:0, PE 14:0/14:0, PE 20:0/20:0 (di-phytanoyl), PS 14:0/14:0, PS 20:0/20:0 (di-phytanoyl), PI 17:0/17:0, LPC 13:0, LPC 19:0, Cer d18:1/14:0, Cer d18:1/17:0, D_7_-FC, CE 17:0, CE 22:0, HexCer d18:1/12:0, HexCer d18:1/17:0, DG 14:0/14:0, DG 20:0/20:0, TG (17:0)_3_, TG (19:0)_3_, and CL (14:0)_4_. Cell homogenates representing approximately 100 μg of protein were subjected to lipid extraction. A total aqueous volume of 800 μl was extracted with 3 ml of methanol/chloroform = 2/1 (v/v) for 1 hour at room temperature. Phase separation was induced by addition of 1 ml each of water and chloroform. Chloroform phase was recovered after centrifugation, dried in a vacuum centrifuge, and dissolved as described below for quantitative lipid analysis.

Lipids were quantified by ESI-MS/MS in positive ion mode as described previously by Ecker and colleagues [45]. In brief, samples were analyzed by direct flow injection using an HTS PAL autosampler (CTC Analytics; Zwingen, Switzerland), an Agilent 1100 binary pump (Agilent Technologies, Waldbronn, Germany), and a triple quadrupole mass spectrometer (Quattro Ultima, Micromass, Manchester, UK)). A precursor ion scan of 184 m/z specific for phosphocholine-containing lipids was used for PC, SM, and LPC [46]. The following neutral losses were applied: PE, 141; PS, 185; PG, 189; and PI, 277 [47]. PE P were analyzed according to the principles described by Berry and colleagues [48]. Sphingosine-based Cers were analyzed using a fragment ion of 264 m/z [49]. FC and CEs were quantified using a fragment ion of 369 m/z after selective derivatization of FC using acetyl chloride [50]. Corrections for isotopic overlap of lipid species and data analysis using Excel Macros were performed for all lipid classes. Non-naturally occurring lipid species were used as internal standards in all analyses. Quantification was performed by the addition of a standard to cell homogenates using a number of naturally occurring lipid species as standards for each lipid class. Lipid species were annotated according to the recently published proposal for shorthand notation of lipid structures derived from MS [51]. Glycerophospholipid species annotation was based on the assumption of even-numbered carbon chains only. SM species annotation is based on the assumption that a sphingoid base with 2 hydroxyl groups is present. Analysis of labeled D_9_-PC and D_9_-LPC upon D_9_-choline labeling was performed as described above using a fragment ion of 193 m/z. Quantification was based on the assumption that labeled and unlabeled species have similar analytical response.

TG, DG, and HexCers were analyzed by HR-MS. Dried lipid extracts were dissolved in chloroform/methanol/2-propanol (1:2:4 v/v/v) with 7.5 mM ammonium formate. The analysis of lipids was performed by direct infusion on a hybrid quadrupole-Orbitrap QExactive mass spectrometer (Thermo Fisher Scientific, Bremen, Germany) equipped with a heated electrospray ionization source. The ion source was operated using the following settings: spray voltage of 3.5 kV, S-lens RF level 50, capillary temperature of 250°C, auxiliary gas heater temperature of 100°C, and flow rates of 15 for sheath gas and 5 for auxiliary gas. All data were acquired in profile mode. A total of 50 μL crude lipid extract of each sample was automatically injected by a PAL system (CTC Analytics, Zwingen, Germany) and infused with a mobile phase chloroform/methanol/2-propanol (1:2:4 v/v/v) at flow rate of 10 μL/min using an UltiMate 3000 isocratic pump (Thermo Fisher Scientific, Bremen, Germany). TG [M+NH4]+, DG [M+NH4]+, and HexCer [M+H]+ were recorded in positive ion mode in the range of 500 to 1,000 m/z for 1 minute with a maximum injection time (IT) of 200 ms, an automated gain control (AGC) of 1 × 10^6^, 3 microscans and a target resolution of 140,000 (at 200 m/z). CLs have been determined as ammoniated adduct in positive ion mode in mass range 1,200 to 1,600 m/z with AGC set to 5 × 10^5^. Lipid species were identified using the ALEX software [52]. Peak assignment applied a mass accuracy of less than 5 ppm. The assigned data were exported to Microsoft Excel 2010 and further processed by self-programmed Macros according to the principles described by Liebisch and colleagues in 2004.

For Cer analysis of BAT, crude lipid extracts were prepared as described above and washed 3 times with ISO-octane to remove TG excess. The remaining fraction was subjected to lipid extraction according to Bligh and Dyer [44] and analyzed as described before. More details, including the internal standards used for quantification, can be found in S1 Data.

For investigation of palmitate metabolism and FBS FA composition FA methyl esters (FAMEs) were generated by acetyl chloride and methanol treatment and extracted with hexan [53]. Total FA analysis was carried out using a Shimadzu 2010 GC-MS system (Duisburg, Germany). FAMEs were separated on a BPX70 column (10 m length, 0.10 mm diameter, 0.20 μm film thickness) from SGE using helium as the carrier gas. The initial oven temperature was 50°C and was programed to increase at 40°C/min to 155°C, 6°C/min to 210°C, and finally 15°C/min to 250°C. U-^13^C-palmitate (Larodan, Solna, Sweden), its desaturation, and elongation products were quantified by single-ion monitoring of specific fragment ions (U-^13^C-palmitate, 286 m/z; U-^13^C-palmitatoleate, 252 m/z; U-^13^C-stearate, 314 m/z). The internal standard was C21:0-iso.

## Supporting information

S1 Fig Common membrane lipid levels; LPC, SM, and CE composition of white, brite, and brown adipocytes. (A) Common membrane lipid levels, (B) LPC composition, (C) SM composition, (D) CE composition. Shown are means ± SD of 3 independent experiments, each performed in triplicates with AT pooled from 3 mice; annotation of “a, b” indicates that group "a" is statistically different from "b"; annotation of “a, b, c” indicates that all 3 groups are significantly different from each other; significant difference was tested using a one-way ANOVA (Post Hoc: Tukey Test; *p* < 0.05). The underlying data of (A–D) can be found in S1 Data. AT, adipose tissue; CE, cholesterylester; LPC, lyso-PC; SM, sphingomyelin.
(TIF)

S2 Fig Effects of β-adrenergic stimulation in white, brite, and brown adipocytes. (A) Lipid class composition of major membrane lipids, (B) LPC/PC, prim. cells; (C) LPC, (D) LPC/PC, prim. brown WT and UCP1 KO adipocytes. Cells were treated for 0.5 hours with 0.5 μM ISO. Shown are means ± SD of 3 mice, “a, b” indicate significant a significant difference between “a” and “b”; determined using a Student *t* test. (E) LPC-WT, (F) LPC-UCP1 KO, (G) LPC/PC-WT, (H) LPC/PC-UCP1 KO, immortalized brite adipocytes, (I) LPC-WT, (J) LPC-UCP1 KO, (K) LPC/PC-WT, (L) LPC/PC-UCP1 KO immortalized brown adipocytes. Cells were treated for 0.5 to 4 hours with 0.5 μM ISO. Shown are means ± SD of 3 replicates, (A–B) annotation of “a, b” indicates that group "a" is statistically different from "b"; (C–L) the *p*-value indicates a significant difference between the treatment groups “Co” and “ISO”; significant difference was tested using a two-way ANOVA (Post Hoc: Tukey Test). The underlying data of (A–L) can be found in S1 Data. Co, control; ISO, isoproterenol; KO, knock out; LPC, lyso-PC; PC, phosphatidylcholine; prim., primary; UCP1, uncoupling protein; WT, wild type.
(TIF)

S1 Table Total FA levels (mmol/l) of 2 human plasma samples [53] and FBS that was added to the cell culture medium. FA, fatty acid; FBS, fetal bovine serum; N.d., not detected.
(DOCX)

S2 Table Comparison of our cellular data with published data from total tissue samples. Adipoc., adipocytes; conc., concentration.
(DOCX)

S1 Data The underlying data of Fig 1A, Fig 2A–2H, Fig 3A–3D, Fig 4A–4F, Fig 5A–5R, Fig 6A–6D, S1A–S1D Fig, and S2A–S2L Fig. (XLSX)

## Abbreviations

Adcy5: adenylate cyclase 5
Adrb3: adrenergic receptor beta 3
AGC: automated gain control
Anti A: antimycin A
AT: adipose tissue
ATGL: adipose triglyceride lipase
BAT: brown adipose tissue
BEL: bromoenollactone
BSA: bovine serum albumin
CE: cholesterylester
Cer: ceramide
CGI-58: comparative gene identification-58
Cidea: cell death-inducing DNA fragmentation factor alpha subunit-like effector A
CL: cardiolipin
Co: control
Cox7a1: cytochrome c oxidase subunit 7A1
Cpt1b: carnitine palmitoyltransferase 1b
DG: diacylglyerol
Dio2: deiodinase iodothyronine type II
Dkl1: delta-like noncanonical Notch ligand 1
ESI-MS/MS: electrospray ionization coupled to tandem mass spectrometry
eWAT: epididymal WAT
FA: fatty acid
Fabp4: fatty acid binding protein 4
FAME: fatty acid methyl ester
FBS: fetal bovine serum
FC: free cholesterol
FCCP: carbonyl cyanide 4-(trifluoromethoxy) phenylhydrazone
FDG: fluorodeoxyglucose
GL: glycerolipid
GPL: glycerophospholipid
HexCer: hexosylceramide
HR-MS: high-resolution mass spectrometry
HSL: hormone-sensitive lipase
iBAT: intrascapular BAT
ISO: isoproterenol
IT: injection time
iWAT: inguinal WAT
KO: knock out
LDH: lactate dehydrogenase
Lep: leptin
LPC: lyso-PC
MS/MS: tandem mass spectrometry
MUFA: monosaturated fatty acid
OCR: oxygen consumption rate
Oligo: oligomycin
PC: phosphatidylcholine
PC O: ether bond-containing PC
PE: phosphatidylethanolamine
PE P: PE-based plasmalogen
PET-CT: positron emission tomography–computed tomography
PI: phosphatidylinositol
PLA_2_: phospholipase A_2_
PS: phosphatidylserine
PUFA: polyunsaturated fatty acid
ROS: reactive oxygen species
RT-PCR: real-time PCR
SAFA: saturated fatty acid
SL: sphingolipid
SM: sphingomyelin
sWAT: subcutanous WAT
Tbx1: T-box 1
Tcf21: transcription factor 21
Tfap2a: transcription factor AP-2 alpha
Tf2b: transcription factor II B
TG: triacylglycerol
Tnfrsf9: tumor necrosis factor receptor superfamily member 9
UCP: uncoupling protein
WAT: white AT
WT: wild type
Zic1: zinc finger protein of the cerebellum 1

## References

[1] Cinti S. The adipose organ: Kurtis Milan; 1999.

[2] Lin CS, Klingenberg M. Isolation of the uncoupling protein from brown adipose tissue mitochondria. FEBS Lett. 1980;113(2):299–303. Epub 1980/05/05. 10.1016/0014-5793(80)80613-2 . 7389900

[3] Petrovic N, Walden TB, Shabalina IG, Timmons JA, Cannon B, Nedergaard J. Chronic peroxisome proliferator-activated receptor gamma (PPARgamma) activation of epididymally derived white adipocyte cultures reveals a population of thermogenically competent, UCP1-containing adipocytes molecularly distinct from classic brown adipocytes. J Biol Chem. 2010;285(10):7153–64. Epub 2009/12/24. 10.1074/jbc.M109.053942 20028987 PMC2844165

[4] Schulz TJ, Huang TL, Tran TT, Zhang H, Townsend KL, Shadrach JL, et al Identification of inducible brown adipocyte progenitors residing in skeletal muscle and white fat. Proc Natl Acad Sci U S A. 2011;108(1):143–8. Epub 2010/12/22. 10.1073/pnas.1010929108 21173238 PMC3017184

[5] Wu J, Boström P, Sparks Lauren M, Ye L, Choi Jang H, Giang A-H, et al Beige Adipocytes Are a Distinct Type of Thermogenic Fat Cell in Mouse and Human. Cell. 2012;150(2):366–76. 10.1016/j.cell.2012.05.016 22796012 PMC3402601

[6] Young P, Arch JR, Ashwell M. Brown adipose tissue in the parametrial fat pad of the mouse. FEBS Lett. 1984;167(1):10–4. 10.1016/0014-5793(84)80822-4 . 6698197

[7] Rosenwald M, Perdikari A, Rulicke T, Wolfrum C. Bi-directional interconversion of brite and white adipocytes. Nature cell biology. 2013;15(6):659–67. Epub 2013/04/30. 10.1038/ncb2740 . 23624403

[8] van Meer G, Voelker DR, Feigenson GW. Membrane lipids: where they are and how they behave. Nat Rev Mol Cell Biol. 2008;9(2):112–24. 10.1038/nrm2330 . 18216768 PMC2642958

[9] Ernst R, Ejsing CS, Antonny B. Homeoviscous Adaptation and the Regulation of Membrane Lipids. J Mol Biol. 2016;428(24 Pt A):4776–91. 10.1016/j.jmb.2016.08.013 . 27534816

[10] Pinot M, Vanni S, Pagnotta S, Lacas-Gervais S, Payet LA, Ferreira T, et al Lipid cell biology. Polyunsaturated phospholipids facilitate membrane deformation and fission by endocytic proteins. Science. 2014;345(6197):693–7. 10.1126/science.1255288 . 25104391

[11] Ecker J, Liebisch G, Englmaier M, Grandl M, Robenek H, Schmitz G. Induction of fatty acid synthesis is a key requirement for phagocytic differentiation of human monocytes. Proc Natl Acad Sci U S A. 2010;107(17):7817–22. 10.1073/pnas.0912059107 . 20385828 PMC2867858

[12] Hermansson M, Hokynar K, Somerharju P. Mechanisms of glycerophospholipid homeostasis in mammalian cells. Prog Lipid Res. 2011;50(3):240–57. 10.1016/j.plipres.2011.02.004 . 21382416

[13] Hoene M, Li J, Haring HU, Weigert C, Xu G, Lehmann R. The lipid profile of brown adipose tissue is sex-specific in mice. Biochim Biophys Acta. 2014;1842(10):1563–70. Epub 2014/08/17. 10.1016/j.bbalip.2014.08.003 . 25128765

[14] May FJ, Baer LA, Lehnig AC, So K, Chen EY, Gao F, et al Lipidomic Adaptations in White and Brown Adipose Tissue in Response to Exercise Demonstrate Molecular Species-Specific Remodeling. Cell Rep. 2017;18(6):1558–72. Epub 2017/02/09. 10.1016/j.celrep.2017.01.038 . 28178530 PMC5558157

[15] Marcher AB, Loft A, Nielsen R, Vihervaara T, Madsen JG, Sysi-Aho M, et al RNA-Seq and Mass-Spectrometry-Based Lipidomics Reveal Extensive Changes of Glycerolipid Pathways in Brown Adipose Tissue in Response to Cold. Cell Rep. 2015;13(9):2000–13. Epub 2015/12/03. 10.1016/j.celrep.2015.10.069 . 26628366

[16] Lynes MD, Shamsi F, Sustarsic EG, Leiria LO, Wang CH, Su SC, et al Cold-Activated Lipid Dynamics in Adipose Tissue Highlights a Role for Cardiolipin in Thermogenic Metabolism. Cell Rep. 2018;24(3):781–90. 10.1016/j.celrep.2018.06.073 . 30021173 PMC6117118

[17] Liaw L, Prudovsky I, Koza RA, Anunciado-Koza RV, Siviski ME, Lindner V, et al Lipid Profiling of In Vitro Cell Models of Adipogenic Differentiation: Relationships With Mouse Adipose Tissues. J Cell Biochem. 2016;117(9):2182–93. 10.1002/jcb.25522 26910604 PMC4957144

[18] Balaz M, Becker AS, Balazova L, Straub L, Muller J, Gashi G, et al Inhibition of Mevalonate Pathway Prevents Adipocyte Browning in Mice and Men by Affecting Protein Prenylation. Cell Metab. 2019;29(4):901–16 e8. 10.1016/j.cmet.2018.11.017 . 30581121

[19] Li YG, Lasar D, Fromme T, Klingenspor M. White, brite, and brown adipocytes: the evolution and function of a heater organ in mammals. Canadian Journal of Zoology. 2014;92(7):615–26. 10.1139/cjz-2013-0165 WOS:000344954300006.

[20] Sun W, Dong H, Becker AS, Dapito DH, Modica S, Grandl G, et al Cold-induced epigenetic programming of the sperm enhances brown adipose tissue activity in the offspring. Nat Med. 2018;24(9):1372–83. 10.1038/s41591-018-0102-y . 29988127

[21] Li Y, Fromme T, Schweizer S, Schottl T, Klingenspor M. Taking control over intracellular fatty acid levels is essential for the analysis of thermogenic function in cultured primary brown and brite/beige adipocytes. EMBO Rep. 2014;15(10):1069–76. 10.15252/embr.201438775 25135951 PMC4253847

[22] Balsinde J, Bianco ID, Ackermann EJ, Conde-Frieboes K, Dennis EA. Inhibition of calcium-independent phospholipase A2 prevents arachidonic acid incorporation and phospholipid remodeling in P388D1 macrophages. Proc Natl Acad Sci U S A. 1995;92(18):8527–31. 10.1073/pnas.92.18.8527 7667324 PMC41190

[23] Li Y, Bolze F, Fromme T, Klingenspor M. Intrinsic differences in BRITE adipogenesis of primary adipocytes from two different mouse strains. Biochimica et Biophysica Acta (BBA)—Molecular and Cell Biology of Lipids. 2014;(0). 10.1016/j.bbalip.2014.06.003. 24953778

[24] Cinti S. The adipose organ. Prostaglandins Leukot Essent Fatty Acids. 2005;73(1):9–15. Epub 2005/06/07. 10.1016/j.plefa.2005.04.010 . 15936182

[25] Reiss D, Beyer K, Engelmann B. Delayed oxidative degradation of polyunsaturated diacyl phospholipids in the presence of plasmalogen phospholipids in vitro. Biochem J. 1997;323 (Pt 3):807–14. 10.1042/bj3230807 9169616 PMC1218386

[26] Furukawa S, Fujita T, Shimabukuro M, Iwaki M, Yamada Y, Nakajima Y, et al Increased oxidative stress in obesity and its impact on metabolic syndrome. J Clin Invest. 2004;114(12):1752–61. 10.1172/JCI21625 15599400 PMC535065

[27] Oelkrug R, Kutschke M, Meyer CW, Heldmaier G, Jastroch M. Uncoupling protein 1 decreases superoxide production in brown adipose tissue mitochondria. Journal of Biological Chemistry. 2010 10.1074/jbc.M110.122861 20466728 PMC2903373

[28] Han XL, Gross RW. Plasmenylcholine and phosphatidylcholine membrane bilayers possess distinct conformational motifs. Biochemistry. 1990;29(20):4992–6. 10.1021/bi00472a032 . 2364071

[29] Paltauf F. Ether lipids in biomembranes. Chem Phys Lipids. 1994;74(2):101–39. . 7859340 10.1016/0009-3084(94)90054-x

[30] Chaurasia B, Kaddai VA, Lancaster GI, Henstridge DC, Sriram S, Galam DL, et al Adipocyte Ceramides Regulate Subcutaneous Adipose Browning, Inflammation, and Metabolism. Cell Metab. 2016;24(6):820–34. 10.1016/j.cmet.2016.10.002 . 27818258

[31] Grosch S, Schiffmann S, Geisslinger G. Chain length-specific properties of ceramides. Progress in lipid research. 2012;51(1):50–62. 10.1016/j.plipres.2011.11.001 . 22133871

[32] Zimmermann R, Strauss JG, Haemmerle G, Schoiswohl G, Birner-Gruenberger R, Riederer M, et al Fat mobilization in adipose tissue is promoted by adipose triglyceride lipase. Science. 2004;306(5700):1383–6. 10.1126/science.1100747 . 15550674

[33] Cannon B, Nedergaard J. Brown adipose tissue: function and physiological significance. Physiological reviews. 2004;84(1):277–359. Epub 2004/01/13. 10.1152/physrev.00015.2003 . 14715917

[34] Takasu N, Hashimoto H, Miyazaki Y, Ito T, Ogawa K, Satake T. Effects of phospholipase inhibitors and calcium antagonists on the changes in myocardial phospholipids induced by isoproterenol. Basic Res Cardiol. 1988;83(5):567–75. . 3233096 10.1007/BF01906686

[35] Schreiber R, Diwoky C, Schoiswohl G, Feiler U, Wongsiriroj N, Abdellatif M, et al Cold-Induced Thermogenesis Depends on ATGL-Mediated Lipolysis in Cardiac Muscle, but Not Brown Adipose Tissue. Cell Metab. 2017;26(5):753–63.e7. Epub 2017/10/11. 10.1016/j.cmet.2017.09.004 28988821 PMC5683855

[36] Shin H, Ma Y, Chanturiya T, Cao Q, Wang Y, Kadegowda AKG, et al Lipolysis in Brown Adipocytes Is Not Essential for Cold-Induced Thermogenesis in Mice. Cell Metab. 2017 Epub 2017/10/11. 10.1016/j.cmet.2017.09.002 . 28988822 PMC5905336

[37] Cannon B, Nedergaard J. What Ignites UCP1? Cell metabolism. 2017;26(5):697–8. 10.1016/j.cmet.2017.10.012 . 29117542

[38] Fedorenko A, Lishko PV, Kirichok Y. Mechanism of fatty-acid-dependent UCP1 uncoupling in brown fat mitochondria. Cell. 2012;151(2):400–13. Epub 2012/10/16. 10.1016/j.cell.2012.09.010 23063128 PMC3782081

[39] Burke JE, Dennis EA. Phospholipase A(2) structure/function, mechanism, and signaling. J Lipid Res. 2009;50(Suppl):S237–42. 10.1194/jlr.R800033-JLR200 19011112 PMC2674709

[40] Dennis EA, Cao J, Hsu YH, Magrioti V, Kokotos G. Phospholipase A2 enzymes: physical structure, biological function, disease implication, chemical inhibition, and therapeutic intervention. Chem Rev. 2011;111(10):6130–85. 10.1021/cr200085w 21910409 PMC3196595

[41] Boon MR, Bakker LEH, Prehn C, Adamski J, Vosselman MJ, Jazet IM, et al LysoPC-acyl C16:0 is associated with brown adipose tissue activity in men. Metabolomics. 2017;13(5). 10.1007/s11306-017-1185-z 28316560 PMC5334436

[42] Klein J, Fasshauer M, Klein HH, Benito M, Kahn CR. Novel adipocyte lines from brown fat: a model system for the study of differentiation, energy metabolism, and insulin action. Bioessays. 2002;24(4):382–8. 10.1002/bies.10058 . 11948624

[43] Yang F, Zhang Y, Liang H. Interactive association of drugs binding to human serum albumin. Int J Mol Sci. 2014;15(3):3580–95. 10.3390/ijms15033580 24583848 PMC3975355

[44] Bligh EG, Dyer WJ. A rapid method of total lipid extraction and purification. Can J Biochem Physiol. 1959;37(8):911–7. 10.1139/o59-099 . 13671378

[45] Ecker J, Liebisch G, Scherer M, Schmitz G. Differential effects of conjugated linoleic acid isomers on macrophage glycerophospholipid metabolism. J Lipid Res. 2010;51(9):2686–94. 10.1194/jlr.M007906 . 20522602 PMC2918450

[46] Liebisch G, Lieser B, Rathenberg J, Drobnik W, Schmitz G. High-throughput quantification of phosphatidylcholine and sphingomyelin by electrospray ionization tandem mass spectrometry coupled with isotope correction algorithm. Biochim Biophys Acta. 2004;1686(1–2):108–17. 10.1016/j.bbalip.2004.09.003 . 15522827

[47] Matyash V, Liebisch G, Kurzchalia TV, Shevchenko A, Schwudke D. Lipid extraction by methyl-tert-butyl ether for high-throughput lipidomics. J Lipid Res. 2008;49(5):1137–46. 10.1194/jlr.D700041-JLR200 18281723 PMC2311442

[48] Zemski Berry KA, Murphy RC. Electrospray ionization tandem mass spectrometry of glycerophosphoethanolamine plasmalogen phospholipids. J Am Soc Mass Spectrom. 2004;15(10):1499–508. 10.1016/j.jasms.2004.07.009 . 15465363

[49] Liebisch G, Drobnik W, Reil M, Trumbach B, Arnecke R, Olgemoller B, et al Quantitative measurement of different ceramide species from crude cellular extracts by electrospray ionization tandem mass spectrometry (ESI-MS/MS). J Lipid Res. 1999;40(8):1539–46. . 10428992

[50] Liebisch G, Binder M, Schifferer R, Langmann T, Schulz B, Schmitz G. High throughput quantification of cholesterol and cholesteryl ester by electrospray ionization tandem mass spectrometry (ESI-MS/MS). Biochim Biophys Acta. 2006;1761(1):121–8. 10.1016/j.bbalip.2005.12.007 . 16458590

[51] Liebisch G, Vizcaino JA, Kofeler H, Trotzmuller M, Griffiths WJ, Schmitz G, et al Shorthand notation for lipid structures derived from mass spectrometry. J Lipid Res. 2013;54(6):1523–30. 10.1194/jlr.M033506 23549332 PMC3646453

[52] Husen P, Tarasov K, Katafiasz M, Sokol E, Vogt J, Baumgart J, et al Analysis of lipid experiments (ALEX): a software framework for analysis of high-resolution shotgun lipidomics data. PloS one. 2013;8(11):e79736 10.1371/journal.pone.0079736 24244551 PMC3820610

[53] Ecker J, Scherer M, Schmitz G, Liebisch G. A rapid GC-MS method for quantification of positional and geometric isomers of fatty acid methyl esters. J Chromatogr B Analyt Technol Biomed Life Sci. 2012;897:98–104. 10.1016/j.jchromb.2012.04.015 . 22542399

