# Supplementary figures and images for: The lipidome of primary murine white, brite, and brown adipocytes—Impact of beta-adrenergic stimulation
Source: PLoS Biol. 2019 Aug 1;17(8):e3000412. doi: 10.1371/journal.pbio.3000412 (PMC6692052; doi:10.1371/journal.pbio.3000412)

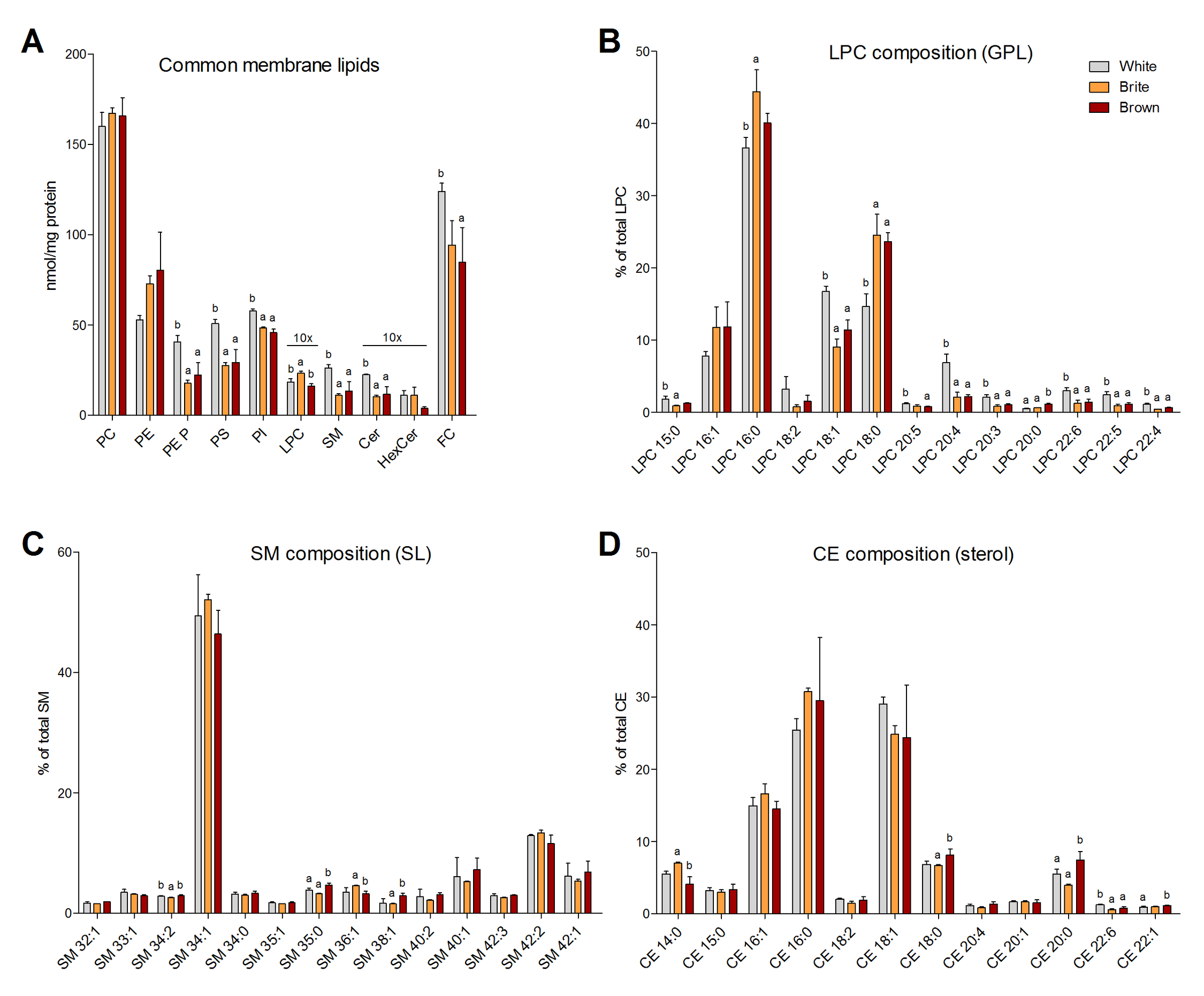

Supplement: S1 Fig — (A) Common membrane lipid levels, (B) LPC composition, (C) SM composition, (D) CE composition. Shown are means ± SD of 3 independent experiments, each performed in triplicates with AT pooled from 3 mice; annotation of “a, b” indicates that group "a" is statistically different from "b"; annotation of “a, b, c” indicates that all 3 groups are significantly different from each other; significant difference was tested using a one-way ANOVA (Post Hoc: Tukey Test; p < 0.05). The underlying data of (A–D) can be found in S1 Data. AT, adipose tissue; CE, cholesterylester; LPC, lyso-PC; SM, sphingomyelin. (TIF) [file pbio.3000412.s001.tif]

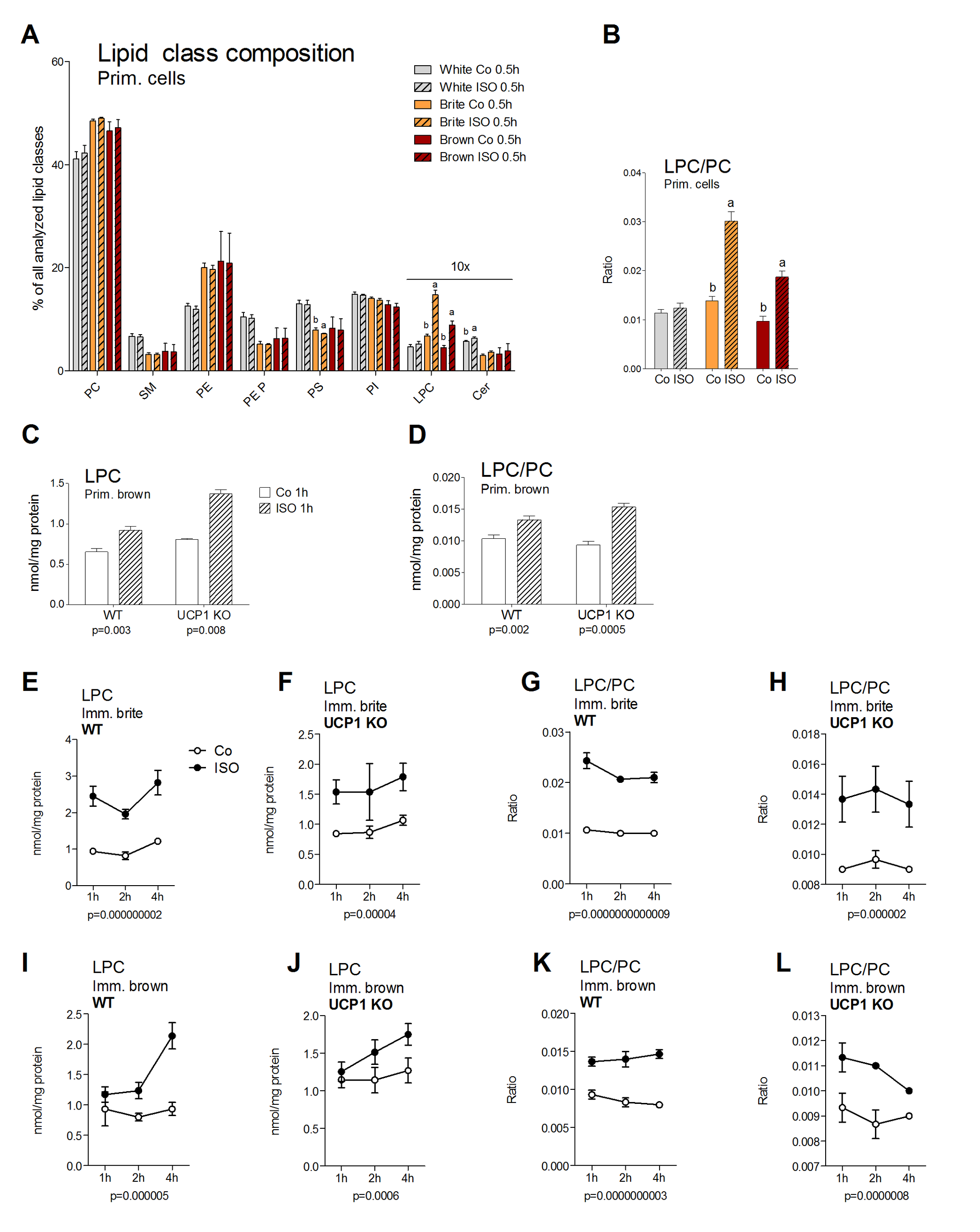

Supplement: S2 Fig — (A) Lipid class composition of major membrane lipids, (B) LPC/PC, prim. cells; (C) LPC, (D) LPC/PC, prim. brown WT and UCP1 KO adipocytes. Cells were treated for 0.5 hours with 0.5 μM ISO. Shown are means ± SD of 3 mice, “a, b” indicate significant a significant difference between “a” and “b”; determined using a Student t test. (E) LPC-WT, (F) LPC-UCP1 KO, (G) LPC/PC-WT, (H) LPC/PC-UCP1 KO, immortalized brite adipocytes, (I) LPC-WT, (J) LPC-UCP1 KO, (K) LPC/PC-WT, (L) LPC/PC-UCP1 KO immortalized brown adipocytes. Cells were treated for 0.5 to 4 hours with 0.5 μM ISO. Shown are means ± SD of 3 replicates, (A–B) annotation of “a, b” indicates that group "a" is statistically different from "b"; (C–L) the p-value indicates a significant difference between the treatment groups “Co” and “ISO”; significant difference was tested using a two-way ANOVA (Post Hoc: Tukey Test). The underlying data of (A–L) can be found in S1 Data. Co, control; ISO, isoproterenol; KO, knock out; LPC, lyso-PC; PC, phosphatidylcholine; prim., primary; UCP1, uncoupling protein; WT, wild type. (TIF) [file pbio.3000412.s002.tif]
