# Supplementary material for: The lipidome of primary murine white, brite, and brown adipocytes—Impact of beta-adrenergic stimulation
Source: PLoS Biol. 2019 Aug 1;17(8):e3000412. doi: 10.1371/journal.pbio.3000412 (PMC6692052; doi:10.1371/journal.pbio.3000412)
Supplement: S1 Table — FA, fatty acid; FBS, fetal bovine serum; N.d., not detected. (DOCX) [file pbio.3000412.s003.docx]

| *FA species* | *Plasma 1* | *Plasma 2* | *FBS (mean, n=4)* | *FBS (SD)* |
| --- | --- | --- | --- | --- |
| FA 10:0 | 0.06 | 0.02 | 0.00 | 0.00 |
| FA 12:0 | 0.01 | 0.05 | 0.21 | 0.03 |
| FA 14:0 | 0.09 | 0.41 | 0.70 | 0.11 |
| FA 15:0 | 0.02 | 0.06 | 0.28 | 0.04 |
| FA 16:0 | 2.18 | 10.67 | 6.05 | 1.10 |
| FA 16:1 *n-7* | 0.16 | 1.30 | 0.56 | 0.15 |
| FA 17:0 | 0.03 | 0.07 | 0.21 | 0.03 |
| FA 18:0 | 0.65 | 2.43 | 2.68 | 0.37 |
| FA 18:1 *n-9* | 1.89 | 10.05 | 2.66 | 0.56 |
| FA 18:1 *n-7* | 0.12 | 0.87 | 0.55 | 0.10 |
| FA 18:2 *n-6* | 1.88 | 7.76 | 0.59 | 0.12 |
| FA 18:3 *n-3* | 0.02 | 0.06 | 0.05 | 0.01 |
| FA 18:3 *n-6* | 0.06 | 0.28 | 0.00 | 0.00 |
| FA 20:0 | 0.01 | 0.05 | 0.07 | 0.02 |
| FA 20:1 *n-9* | 0.01 | 0.09 | 0.07 | 0.01 |
| FA 20:3 n-6 | 0.07 | 0.38 | 0.19 | 0.03 |
| FA 20:4 *n-6* | 0.32 | 1.24 | 0.72 | 0.14 |
| FA 20:3 *n-3* | n.d. | n.d. | 0.02 | 0.02 |
| FA 20:4 *n-3* | n.d. | n.d. | 0.04 | 0.01 |
| FA 22:0 | 0.02 | 0.08 | 0.10 | 0.02 |
| FA 20:5 *n-3* | 0.04 | 0.07 | 0.12 | 0.02 |
| FA 22:4 *n-6* | 0.01 | 0.05 | 0.00 | 0.00 |
| FA 24:0 | 0.01 | 0.04 | 0.05 | 0.01 |
| FA 24:1 *n-9* | 0.05 | 0.23 | 0.07 | 0.05 |
| FA 22:5 *n-6* | 0.02 | 0.09 | 0.22 | 0.04 |
| FA 22:6 *n-3* | 0.08 | 0.29 | 0.35 | 0.10 |
| SAFA | 3.10 | 13.87 | 10.35 | 1.58 |
| MUFA | 2.22 | 12.53 | 3.92 | 0.79 |
| PUFA | 2.51 | 10.22 | 2.31 | 0.42 |
