# Supplementary material for: The lipidome of primary murine white, brite, and brown adipocytes—Impact of beta-adrenergic stimulation
Source: PLoS Biol. 2019 Aug 1;17(8):e3000412. doi: 10.1371/journal.pbio.3000412 (PMC6692052; doi:10.1371/journal.pbio.3000412)
Supplement: S2 Table — Adipoc., adipocytes; conc., concentration. (DOCX) [file pbio.3000412.s004.docx]

| Reference | Matrix | m/f | conc. unit |  | PC | PE | PE P | PS | PI | LPC | SM | Cer | HexCer | FC | CL | TAG | DAG | CE |
| --- | --- | --- | --- | --- | --- | --- | --- | --- | --- | --- | --- | --- | --- | --- | --- | --- | --- | --- |
| Own Data | White Adipoc./eWAT | m | nmol/mg prot. | Mean | **160.0** | **52.6** | **40.6** | **50.7** | **57.7** | **1.8** | **26.1** | **2.2** | **1.1** | **123.8** | **1.1** | **396.9** | **13.8** | **7.8** |
|  |  |  |  | SD | 7.6 | 2.7 | 3.6 | 2.1 | 1.2 | 2.0 | 1.9 | 0.4 | 2.7 | 4.7 | 0.2 | 188.1 | 4.6 | 0.5 |
|  | Brite Adipoc./iWAT | m | nmol/mg prot. | Mean | **167.2** | **72.7** | **17.8** | **27.3** | **48.4** | **2.3** | **11.0** | **1.0** | **1.1** | **94.3** | **3.9** | **1772.7** | **36.8** | **10.2** |
|  |  |  |  | SD | 2.9 | 4.6 | 1.6 | 1.8 | 0.4 | 1.3 | 1.0 | 0.8 | 4.4 | 13.4 | 0.8 | 176.3 | 4.8 | 0.9 |
|  | Brown Adipoc./iBAT | m | nmol/mg prot. | Mean | **165.9** | **80.1** | **22.2** | **29.3** | **45.7** | **1.6** | **13.5** | **1.2** | **3.8** | **84.7** | **7.9** | **1678.3** | **36.6** | **5.8** |
|  |  |  |  | SD | 10.0 | 21.1 | 6.9 | 7.1 | 2.0 | 1.4 | 5.3 | 4.1 | 1.0 | 19.1 | 0.4 | 24.8 | 2.2 | 0.3 |
|  |  |  |  |  |  |  |  |  |  |  |  |  |  |  |  |  |  |  |
| Hoene 2014 | gWAT (→ eWAT) | m | nmol/mg | Mean | **1.4** | **0.6** | **0.2** | **0.4** | **0.1** | **0.1** | **0.2** | **0.1** | **0.01** | **-** | **-** | **233.1** | **0.3** | **-** |
| [1] |  |  |  | SD | 0.2 | 0.1 | 0.0 | 0.1 | 0.0 | 0.0 | 0.0 | 0.0 | 0.00 |  |  | 17.5 | 0.0 |  |
|  | sWAT (→ iWAT) | m | nmol/mg | Mean | **2.2** | **1.2** | **0.6** | **0.7** | **0.3** | **0.1** | **0.3** | **0.1** | **0.04** | **-** | **-** | **189.0** | **0.3** | **-** |
|  |  |  |  | SD | 0.4 | 0.3 | 0.1 | 0.1 | 0.1 | 0.0 | 0.0 | 0.0 | 0.01 |  |  | 30.6 | 0.1 |  |
|  | BAT | m | nmol/mg | Mean | **6.8** | **9.8** | **2.1** | **0.8** | **1.1** | **0.3** | **0.4** | **0.1** | **0.02** | **-** | **-** | **110.8** | **0.3** | **-** |
|  |  |  |  | SD | 1.0 | 1.1 | 0.2 | 0.1 | 0.1 | 0.1 | 0.0 | 0.0 | 0.01 |  |  | 11.9 | 0.1 |  |
|  |  |  |  |  |  |  |  |  |  |  |  |  |  |  |  |  |  |  |
| May 2017 | sWAT (→ iWAT) | m | nmol/mg prot. | Mean | **88.7** | **22.8** | **1.8** | **23.9** | **3.2** | **3.9** | **23.2** | **1.5** | **2.9** | **-** | **9.4** | **4452.1** | **208.9** | **0.8** |
| [2] |  |  |  | SD | 9.0 | 3.9 | 0.2 | 9.0 | 0.9 | 0.8 | 6.0 | 0.3 | 1.0 |  | 2.7 | 564.1 | 34.7 | 0.9 |
|  | BAT | m | nmol/mg prot. | Mean | **93.0** | **44.6** | **3.4** | **30.3** | **3.7** | **4.9** | **13.5** | **0.5** | **0.9** | **-** | **21.3** | **1694.9** | **81.5** | **0.2** |
|  |  |  |  | SD | 12.6 | 17.6 | 1.2 | 6.6 | 0.7 | 1.9 | 5.4 | 0.3 | 0.1 |  | 4.1 | 372.5 | 17.8 | 0.1 |
|  |  |  |  |  |  |  |  |  |  |  |  |  |  |  |  |  |  |  |
| Lynes 2018 | sWAT (→ iWAT) | m | nmol/mg prot. | Mean | **113.6** | **26.0** | **2.3** | **15.4** | **2.3** | **2.9** | **84.9** | **1.3** | **6.6** | **-** | **4.3** | **3347.3** | **99.4** | **0.1** |
| [3] |  |  |  | SD | 19.8 | 4.1 | 0.5 | 2.6 | 0.4 | 0.7 | 57.9 | 0.4 | 3.1 |  | 0.8 | 979.4 | 39.9 | 0.1 |
|  | BAT | m | nmol/mg prot. | Mean | **155.8** | **74.3** | **6.9** | **47.5** | **3.4** | **4.6** | **12.2** | **0.5** | **1.6** | **-** | **16.9** | **687.6** | **35.4** | **0.2** |
|  |  |  |  | SD | 46.9 | 10.5 | 1.1 | 9.7 | 0.4 | 0.6 | 5.7 | 0.1 | 0.4 |  | 2.0 | 61.6 | 5.9 | 0.2 |

References:

1. Hoene M, Li J, Haring HU, Weigert C, Xu G, Lehmann R. The lipid profile of brown adipose tissue is sex-specific in mice. Biochim Biophys Acta. 2014;1842(10):1563-70. Epub 2014/08/17. doi: 10.1016/j.bbalip.2014.08.003. PubMed PMID: 25128765.
2. May FJ, Baer LA, Lehnig AC, So K, Chen EY, Gao F, et al. Lipidomic Adaptations in White and Brown Adipose Tissue in Response to Exercise Demonstrate Molecular Species-Specific Remodeling. Cell Rep. 2017;18(6):1558-72. Epub 2017/02/09. doi: 10.1016/j.celrep.2017.01.038. PubMed PMID: 28178530.
3. Lynes MD, Shamsi F, Sustarsic EG, Leiria LO, Wang CH, Su SC, et al. Cold-Activated Lipid Dynamics in Adipose Tissue Highlights a Role for Cardiolipin in Thermogenic Metabolism. Cell Rep. 2018;24(3):781-90. doi: 10.1016/j.celrep.2018.06.073. PubMed PMID: 30021173.
